# Risk Factors and Early Predictors for Heterotopic Pregnancy after In Vitro Fertilization

**DOI:** 10.1371/journal.pone.0139146

**Published:** 2015-10-28

**Authors:** Meiju Liu, Xiuqing Zhang, Ling Geng, Mingdi Xia, Junyu Zhai, Wei Zhang, Yuchao Zhang, Yinhua Sun, Jiangtao Zhang, Dongyi Zhu, Han Zhao, Zi-Jiang Chen

**Affiliations:** 1 Center for Reproductive Medicine, Provincial Hospital Affiliated to Shandong University, Jinan 250001, China; 2 National Research Center for Assisted Reproductive Technology and Reproductive Genetic, Jinan, China; 3 The Key laboratory for Reproductive Endocrinology of Ministry of Education, Jinan, China; 4 Shandong Provincial Key Laboratory of Reproductive Medicine, Jinan 250021, China; 5 Department of Reproductive Medicine, Linyi People’s Hospital, Linyi 276000, China; 6 Department of joint and bone oncology, Provincial Hospital Affiliated to Shandong University, Jinan 250001, China; 7 Center for Reproductive Medicine, Renji Hospital, School of Medicine, Shanghai Jiaotong University; Shanghai Key Laboratory for Assisted Reproduction and Reproductive Genetics, Shanghai 200135, China; Institute of Zoology, Chinese Academy of Sciences, CHINA

## Abstract

This study investigated the risk factors and early predictors for heterotopic pregnancy (HP) after in vitro fertilization and embryo transfer (IVF-ET). From January 2008 to January 2013, 41 cases of HP and 72 cases of intrauterine twin pregnancy after IVF-ET were recruited and retrospectively analyzed. Compared with intrauterine twin pregnancy group, the HP group had a lower basal luteinizing hormone (LH) level (*P* = 0.005) and more cases had a history of hydrosalpinx (*P* = 0.008). After 14 days of IVF-ET, the serum β-HCG (β-human chorionic gonadotropin), E2 (Estradiol) and P (Progesterone) levels were lower in HP group (*P*<0.001, respectively). Moreover, vaginal bleeding and abdominal pain were the significant features of HP before diagnosis (*P*<0.001, respectively). Further by logistic regression, serum β-hCG, P levels on the 14th day after ET, and vaginal bleeding were identified as the independent factors of HP. These results indicate that when two or more embryos transferred in IVF procedure, β-hCG, P levels on the 14th day after ET, and vaginal bleeding could be taken as predictors for HP.

## Introduction

Heterotopic pregnancy (HP) is defined as co-existence of extra-uterine and intra-uterine gestations, occurring at two or more implantation sites. The incidence of HP in spontaneous pregnancies is about 1/30,000 (1/10,000-1/50,000). In recent years, the incidence of HP is gradually increased, which has reached to 1.5/1000[1], and some areas have even been closed to 1/100 [2]. The difference in these percentages is attributed to the high incidence of pelvic inflammatory disease (PID) and two or more embryos transfer in IVF procedure [2–6]. The diagnosis of HP is often ignored or delayed, which endangers the lives and health of both the affected mother and embryos [7, 8]. In early stage extra-uterine gestation is not easy to be identified by ultrasound, and serum β-hCG level measurement may not be very useful because of the accompanying intra-uterine pregnancy (IUP) [9]. Previous studies have showed the risk factors for HP includes tubal damage after pelvic inflammatory disease, endometriosis, tubal infertility or tubal reconstructive surgery, and uterus malformation, et al, similar to ectopic pregnancy(EP) [10–12]. However, there was short of a definite conclusion for risk factors or early predictors for HP, especially the comparison of intrauterine twin pregnancy and HP, which has never been reported.

The purpose of the study was to evaluate risk factors (tubal infertility, previous ectopic pregnancy, PID, and previous surgery for endometriosis or myomectomy, et al) and to further explore early predictors (vaginal bleeding, abdominal pain, serum β-HCG, E2, P levels and so on) associated with HP among women who conceived IVF-ET. Then, by early diagnosis and timely treatment to prevent serious complications, ensure the continued intrauterine pregnancy, and finally achieve the desired therapeutic effect.

## Materials and Methods

### Study population

We reviewed the medical records of women who conceived treatment for IVF cycles at Center for Reproductive Medicine, Provincial Hospital Affiliated to Shandong University from January 2008 to January 2013 and recruited 41 cases of HP and 72 cases of intrauterine twin pregnancy both using fresh embryos transfer. HP was diagnosed based on serum β-hCG level above 5 mIU/ml after the 14 days of embryo transfer; and β-hCG continued to rise, with or without abdominal pain, vaginal bleeding and other symptoms; then pregnant uterine sac echo mass, extra-uterine gestational sac, pelvic fluid were found by transvaginal B ultrasonic (TVB). Of the 41 cases with HP, 39 were finally diagnosed by laparoscopy and the other 2 cases were diagnosed with extra-uterine heart beats whereas the intra-uterine embryo stopped growth under TVB. Cases of intrauterine twin pregnancy were diagnosed also based on serum β-hCG above 5 mIU/ml after the 14 days of embryo transfer, and two gestational sac and primitive heart tube pulse were found in the uterine cavity by TVB after the 35 days of embryo transfer; the two fetus were normal by abdominal sonography after 65 days of embryo transfer.

### Data collection

Two groups of cases were respectively collected with age, gravidity, relevant medical history of ectopic pregnancy, abortion, chronic pelvic inflammatory disease, tuberculosis treatment, HSG (hysterosalpingography), TVS (Transvaginal Hysteroscopy), TVB and other related test results when the women were interviewed as inpatients. Clinical data included abdominal pain, vaginal bleeding, general appearance on ultrasound (presence of adnexal mass or gestation sac), and location of the HP according to ultrasonography or intra-operative observation, β-hCG, E2, P, et al.

### Embryo transfer and pregnancy detection

After IVF, two embryos were transferred for cases in our study. The transfer location was the distance at the bottom of the uterus 1–1.5cm. Supplementation of early pregnancy was administered with intra-muscular progesterone until 8–10 weeks of gestation. Pregnancy or not was assessed by serum β-hCG levels after the 14 days of embryo transfer. At about 7 weeks of gestation (5 weeks after ET) routine TVB examination was performed among all cases, and repeated ultrasound was executed rapidly if cases presented with symptoms of pain and/or vaginal bleeding at any time. We took surgical or medical treatment for therapeutic initiatives.

### Statistical analysis

Statistical analysis was performed using Statistical Package for the Social Sciences Version 20.0 (IBM Corp, Armonk, NY, USA). Measurement data was compared using Student’s t-test, count data was compared using Student’s χ^2^- test, heterotopic pregnancy and intrauterine twin pregnancy of risk factors and early predictors were compared using Logistic regression analysis. A value of *P* < 0.05 was considered statistically significant.

## Results

General characteristics of HP and intrauterine twin pregnancy cases were compared in S1 Table. There was no significant difference in age, duration of infertility, basal FSH, E2 levels (*P* > 0.05), but there was a significant difference in the level of basal LH between two groups (*P* = 0.005). The level of basal LH was much higher in the cases of intrauterine twin pregnancy.

Possible risk factors for HP were shown in S2 Table. The proportion of cases with preexisting hydrosalpinx was significantly higher in HP group than in intrauterine twin pregnancy group (*P* = 0.008). The respective proportion of induced abortion, tubal adhesions and cervical mycoplasma infection was slightly higher in HP group than those in intrauterine twin pregnancy group, but there was no statistically significant difference (*P* > 0.05).

On the day of HCG administration, E2, LH and P were tested; and before ET endometrial thickness was measured. Only endometrial thickness was slightly thinner in HP group (*P* = 0.077) (S3 Table). However, on the 14^th^ day after ET (ET14d), β-HCG, E2 and P were all lower in HP than those in the controls group (*P*<0.001, respectively). Besides, among the 41 cases with HP, 19 had vaginal bleeding (46.34%) and 15 with abdominal pain(36.6%), whereas the symptoms were rarely appeared in the intrauterine pregnancy cases, with only 2/72(2.78%) vaginal bleeding and 1/72(1.39%) abdominal pain.(S3 Table).

To further identify the independent impact factors of HP, logistic regression analysis was conducted and the serum β-HCG, P on the 14th day of ET, and vaginal bleeding were authenticated as independent predictors of HP (S4 Table).

## Discussion

With widespread using of ART, many problems of infertility were solved, but the incidence of rare diseases increased, such as multiple gestation, EP and HP. In the HP’s cases, EP was usually located in the uterine tubes [13]. Once the tubal pregnancy was ruptured, it would threaten the safety of intrauterine embryo or fetus, even the lives of women. Therefore it was essential to explore the risk factors and predictors for HP after IVF-ET.

Tubal inflammation and mechanical injury have been shown to be risk factors for HP’s cases among women treated with IVF by Brodowska et al. who had studied in 3019 transfer cycles [14]. Casikar et al.also recognized tubal factor infertility appeared to be the risk factor for HP after IVF treatment [15]. In the current study, more cases had a history of hydrosalpinx in the HP group indicated that hydrosalpinx might be a risk factor. Hydrosalpinx might impair tubal function (such as the motility of normal rhythmic contraction and the ciliary movement). So timely treatment of hydrosalpinx before IVF-ET could improve the outcome of IVF treatment, and reduce the incidence of HP and abortion, which had been affirmed by previous studies [16]. In our study we found that the incidence of tubal adhesions, tubal obstruction was higher in HP groups than in intrauterine twin pregnancy group, but with no statistical difference, which might due to the limited number of HP cases.

Pelvic surgery or pelvic infection has been identified as a risk factor for IVF HP by Yu and others [11,17–18]. In our study, the incidence of artificial abortion, cervical mycoplasma infection was higher in HP group in comparison with control group, but there was no significant difference. It is possible that routine examination of genital tract microbes had been done, and pelvic infection had been treated before IVF treatment. This could explain why there was no apparent increase in HP’s women. The independent analysis results showed that neither the history of EP nor the history of chronic pelvic inflammatory disease, appendicitis and so on, appeared to be correlated with IVF HP. The incidence of pelvic injury was higher in HP by consolidating analysis to the factors affecting pelvic environment, but there was no statistically significant difference. In addition, intrauterine factors such as endometritis, endometrial polyps, uterine fibroids, and endometriosis appeared no risk to HP after IVF treatment. In our center, all cases were required to undergo hysteroscope examination and timely treatment of abnormal factors (such as endometrial inflammatory, uterine mediastinum, endometrial polyps) before IVF treatment. That might be why uterine factor was not the risk factor in our study.

Serum E2 level has been identified as a risk factor before ovulation in Verhulst G’s study. They found that serum E2 level was above 3670 pmol/L in more than 90% of the HP cases [19]. However, our study showed that serum E2 level in HP cases (4254.82 ± 2242.20) was above 4000 pmol / L, higher than those in intrauterine twin pregnancy cases (3947.52 ± 2340.66), which was consistent with Verhulst G’s study, but there was no significant difference. In addition, we noted that there were apparent difference of the basal LH levels in intrauterine twin pregnancy cases and in HP cases, but the basal LH levels had not been identified as a risk factor for HP.

Mol et al. [20] reported that endometrial thickness was an indicator for EP. In our study the results showed that the endometria was thinner in HP group than that in intrauterine pregnancy group, but there was no significant difference (*P* = 0.077). It is probably that extra added estrogen and progesterone decreased the effect of this factor.

The measurement of serum quantitative β-hCG levels seemed to have considerable merit in diagnosing EP or pregnancy of unknown location [21]. Sgantha and others suggested that we should be sharp vigilance to multiple pregnancies or HP if serum β-hCG level was above 300 IU / L after the 14 days of IVF-ET [22]. So we requires a high index predictor to strengthen diagnose at early gestational age. In our cases we noticed that the level of serum β-hCG was lower in HP (630.14±362.84IU/L) than in intrauterine twin pregnancy(1227.14±80.83IU/L) on the 14^th^ day after IVF-ET, but was higher than intrauterine single pregnancy(490.93±276.71) in our centers [23]. So we thought the level of serum β-hCG was an important predictor for HP after IVF -ET.

In addition, the level of serum P after the 14^th^ of IVF-ET was another predictor for HP. The results showed that the level of P was lower in HP cases than in intrauterine twin pregnancy cases. So if serum β-hCG level was abnormally higher after the 14^th^ of IVF-ET, accompanied by abnormal P, the probability of HP should be taken into account. The careful reexamination of the adnexa was necessary by an experienced sonographer, which would demonstrate the ectopic pregnancy and the presence of fluid within the pelvis.

It was reported that the incidence of abdominal pain in HP cases was 83%, hypovolemic shock and abdominal tenderness was 13%; half of HP cases experienced vaginal bleeding [13]. The diagnosis of HP is challenging. It is difficult as it is rarely encountered and the presence of intrauterine pregnancy often impedes the diagnosis and early intervention for the EP. Abdominal pain, adnexal mass, peritoneal irritation and an enlarged uterus are four common presenting signs and symptoms in EP women [24]. But HP can present with the signs of either intrauterine pregnancy or EP, or a combination, and about 50% of HP cases have no obvious clinical symptoms [25]. We found that the occurrence of abdominal pain due to peritoneal irritation appeared in 36.59%, vaginal bleeding in 46.34% of HP cases, was obviously higher than control group. After logistic regression, vaginal bleeding was an independent predictor for HP after IVF-ET.

The careful examination or reexamination of the adnexa by a skilled sonographer and the presence of fluid within the pelvis, could guide doctors to a right diagnosis. The high-resolution transvaginal sonography is the most important diagnostic method for intrauterine pregnancy [26].Condous et al reported that 40% to 70% in the cases of HP were checked out by transvaginal ultrasound, and the rest needed the help of laparoscopy [27]. The purpose of the treatment is to preserve the intrauterine pregnant and discontinue the development of the ectopic pregnancy. The first-line option treatment for HP is surgery by laparoscopy or laparotomy. Among the 41 cases with HP, 39 cases were treated with excision of the affected oviduct by laparoscopy and 33 cases (84.6%) had successful deliveries. The other 2 cases were diagnosed with extra-uterine heart beats whereas the intra-uterine embryo stopped growth. Then we suggest that once the HP is diagnosed, timely treatment is essential [28] and we preferred laparoscopy treatment.

In conclusion, this study indicated that the levels of serum β-hCG, P on the 14^th^ day after IVF-ET and vaginal bleeding are independent risk factors for HP.

## Supporting Information

S1 Table General characteristics of HP and intrauterine twin cases. (DOC)

S2 Table Possible pelvic and uterine factors for HP. (DOC)

S3 Table Relevant indicators were analyzed in the two groups. (DOC)

S4 Table Results of risk factors and early predictors in two groups were analyzed by logistic regression analysis. (DOC)
