# Supplementary material for: Risk Factors and Early Predictors for Heterotopic Pregnancy after In Vitro Fertilization
Source: PLoS One. 2015 Oct 28;10(10):e0139146. doi: 10.1371/journal.pone.0139146 (PMC4624796; doi:10.1371/journal.pone.0139146)
Supplement: S1 Table — (DOC) [file pone.0139146.s001.doc]

**S1 Table General characteristics of HP and intrauterine twin cases**

| **Project** | **HP** | **intrauterine twin** | **t** | **p** |
| --- | --- | --- | --- | --- |
| Age (years) | 31.46±4.08 | 30.13±4.30 | 1.62 | 0.108 |
| Infertility (years) | 4.93±2.78 | 3.96±2.38 | 1.95 | 0.054 |
| Basal FSH(IU/L) | 6.82±2.49 | 6.74±1.64 | 0.19 | 0.852 |
| Basal LH(IU/L) | 4.48±1.76 | 6.31±3.85 | -2.87 | **0.005** |
| Basal E2(pg/ml) | 41.66±29.35 | 45.37±20.05 | -0.79 | 0.429 |

FSH: Follicle-Stimulating Hormone ; LH: Luteinizing Hormone ; E2：Estradiol
