# Supplementary material for: Risk Factors and Early Predictors for Heterotopic Pregnancy after In Vitro Fertilization
Source: PLoS One. 2015 Oct 28;10(10):e0139146. doi: 10.1371/journal.pone.0139146 (PMC4624796; doi:10.1371/journal.pone.0139146)
Supplement: S2 Table — (DOC) [file pone.0139146.s002.doc]

**S2 Table Possible pelvic and uterine factors for HP**

| **Medical history** | **HP(N,%)** | **Intrauterine twin(N,%)** | **χ2** | **P** |
| --- | --- | --- | --- | --- |
| Ectopic pregnancy | 7(17.1) | 16(22.2) | 0.427 | 0.513 |
| Artificial abortion | 16(39.0) | 23(31.9) | 0.579 | 0.447 |
| Chronic PID | 0(0) | 0(0) |  |  |
| Appendicitis | 1(2.4) | 0(0) | 1.772 | 0.363* |
| Pelvic surgery | 13(31.7) | 27(37.5) | 0.383 | 0.536 |
| Cervical mycoplasma infection | 4(9.76) | 2(2.78) | 1.333 | 0.248 |
| IUD use | 5(12.1) | 9(12.5) | 0.002 | 0.962 |
| Hydrosalpinx | 12(29.3) | 7(9.7) | 7.136 | **0.008** |
| Tubal adhesions | 14(34.1) | 18(25) | 0.077 | 0.299 |
| Tubal obstruction | 11(26.8) | 17(23.6) | 0.145 | 0.703 |
| Endometritis | 0(0) | 2(2.78) | 1.159 | 0.534* |
| Endometrial polyps | 2(4.87) | 8(11.1) | 0.604 | 0.437 |
| TB treatment | 5(12.2) | 12(16.7) | 0.409 | 0.523 |
| Uterine fibroids | 1(2.4) | 0(0) | 1.772 | 0.363* |
| Endometriosis | 0(0) | 2(2.78) | 1.159 | 0.534* |
| Total cases | 41(100) | 72(100) |  |  |

* Fisher's exact test. PID: pelvic inflammatory disease; IUD: intrauterine device; TB: Tuberculosis
