# Supplementary material for: Risk Factors and Early Predictors for Heterotopic Pregnancy after In Vitro Fertilization
Source: PLoS One. 2015 Oct 28;10(10):e0139146. doi: 10.1371/journal.pone.0139146 (PMC4624796; doi:10.1371/journal.pone.0139146)
Supplement: S3 Table — (DOC) [file pone.0139146.s003.doc]

**S3 Table Relevant indicators were analyzed in the two groups**.

| **Related indicators** | **HP** | **Intrauterine twin** | **SV** | **p** |
| --- | --- | --- | --- | --- |
| E2_-HCG_ (pg/ml) | 4254.82±2242.20 | 3947.52±2340.66 | 0.658 | 0.512 |
| LH_-HCG_ (pg/ml) | 2.61±2.47 | 2.56±2.0 | 0.103 | 0.918 |
| P _-HCG_ (pg/ml) | 0.93±0.56 | 0.99±0.38 | -0.715 | 0.476 |
| EM thickness_-ET_ (mm) | 1.10±0.19 | 1.16±0.12 | -1.799 | 0.077 |
| Serumβ-HCG_-ET14d_ (mIU/ml) | 630.14±362.84 | 1227.14±807.83 | -4.416 | **<0.001** |
| E2_--ET14d_ (pg/ml) | 613.81±589.88 | 1248.48±722.18 | -4.736 | **<0.001** |
| P_-ET14d_ (ng/ml) | 38.97±16.89 | 56.39±11.81 | -6.403 | **<0.001** |
| Vaginal bleeding (n) (%) | 19(46.34%) | 2(2.78%) | 32.767 | **<0.001** |
| Abdominal pain (n) (%) | 15(36.59%) | 1(1.39%) | 26.626 | **<0.001** |

SV: statistical values. –HCG: testing performed on the day of HCG administration;-ET:embryo transfer; -ET14d: testing performed on the 14th day after ET. EM: Endometrial. Abdominal pain and vaginal bleeding were compared with χ2 -testing, others were compared with t-testing.
