# Supplementary material for: Risk Factors and Early Predictors for Heterotopic Pregnancy after In Vitro Fertilization
Source: PLoS One. 2015 Oct 28;10(10):e0139146. doi: 10.1371/journal.pone.0139146 (PMC4624796; doi:10.1371/journal.pone.0139146)
Supplement: S4 Table — (DOC) [file pone.0139146.s004.doc]

**S4 Table Results of risk factors and early predictors in two groups were analyzed by logistic regression analysis.**

| **Risk factors and predictors** | ***P*** | **Relative risk** | **95% CI*** | |
| --- | --- | --- | --- | --- |
| Basal LH | 0.107 | 1.292 | 0.946 | 1.765 |
| Hydrosalpinx | 0.079 | 0.167 | 0.023 | 1.231 |
| β-hCG_-ET14d_ | 0.025 | 1.002 | 1.000 | 1.004 |
| E2_-ET14d_ | 0.383 | 1.001 | 0.999 | 1.002 |
| P_-ET14d_ | 0.002 | 1.088 | 1.032 | 1.146 |
| Abdominal pain | 0.116 | 0.131 | 0.010 | 1.656 |
| Vaginal bleeding | 0.043 | 0.109 | 0.013 | 0.933 |

* 95% confidence intervals. -ET14d: testing performed on the 14th day after ET
